# Fire Ant Venom Alkaloids Inhibit Biofilm Formation

**DOI:** 10.3390/toxins11070420

**Published:** 2019-07-18

**Authors:** Danielle Bruno de Carvalho, Eduardo Gonçalves Paterson Fox, Diogo Gama dos Santos, Joab Sampaio de Sousa, Denise Maria Guimarães Freire, Fabio C. S. Nogueira, Gilberto B. Domont, Livia Vieira Araujo de Castilho, Ednildo de Alcântara Machado

**Affiliations:** 1 Departamento de Parasitologia, Instituto de Biofísica Carlos, Chagas Filho (IBCCF), Universidade Federal do Rio de Janeiro, Rio de Janeiro 21044-020, Brazil; 2 Red Imported Fire Ant Research Centre, South China Agricultural University (SCAU), Guangzhou 510642, China; 3 Departamento de Bioquímica (DBq), Instituto de Química, Universidade Federal do Rio de Janeiro, Rio de Janeiro 21044-020, Brazil

**Keywords:** natural antibiotics, piperidine heterocyclic amines, industrial biotechnology, LTQ Orbitrap Hybrid Mass Spectrometer, myrmecology

## Abstract

Biofilm formation on exposed surfaces is a serious issue for the food industry and medical health facilities. There are many proposed strategies to delay, reduce, or even eliminate biofilm formation on surfaces. The present study focuses on the applicability of fire ant venom alkaloids (aka ‘solenopsins’, from *Solenopsis invicta*) tested on polystyrene and stainless steel surfaces relative to the adhesion and biofilm-formation by the bacterium *Pseudomonas fluorescens*. Conditioning with solenopsins demonstrates significant reduction of bacterial adhesion. Inhibition rates were 62.7% on polystyrene and 59.0% on stainless steel surfaces. In addition, solenopsins drastically reduced cell populations already growing on conditioned surfaces. Contrary to assumptions by previous authors, solenopsins tested negative for amphipathic properties, thus understanding the mechanisms behind the observed effects still relies on further investigation.

## 1. Introduction

Any exposed surface, ranging from biological tissues to stainless steel, is vulnerable to the adhesion of microorganisms, whose accumulated secreted factors may lead to the formation of a biofilm matrix [1,2]. Biofilm formation is a primary cause of urinary tract inflammations, rejection of surgically implanted parts and prostheses, and of dental plaque formation [3,4]. Furthermore, major economic losses associated to biofilm formation can result from biological corrosion of duct pipes and connections, disruption of heat modulators, accelerated material degradation, and food contamination [4,5,6]. As a result, the formation of biofilm is a major health and industrial concern.

When immersed within their secreted biofilm, microorganisms may become thousands of times more resistant to methods of control (e.g., antibiotics, disinfectants) as compared to their free-living state [6,7]. The acquired resistance makes the removal of microorganisms more difficult, which poses a problem in industrial and medical spheres [7,8,9]. The most commonly employed chemicals against biofilm formation are organic halogen compounds, peroxides, inorganic acids, anionic detergents, and surfactants [6]. Because of their high toxicity, low biodegradability, and high surface abrasion [10,11], these chemicals become additional hazards to humans and the environment.

A range of microorganisms can form biofilms, of which the gram-negative bacterium *Pseudomonas fluorescens* (Flügge 1886) is one of the most intensively studied. This aerobic bacterium will rapidly form biofilms on surfaces with different physicochemical properties, including polystyrene, stainless steel, and polyamides [12,13]. Though regarded as non-pathogenic, *P. fluorescens* is often involved in rapid food contamination, leading to gastroenteritis [14,15]. Moreover, *Pseudomonas* spp. are among the most abundant microbes detected in industrial water circuits, and are associated with augmented corrosion and clogging of pipelines, ultimately leading to loss of speed, load capacity, and unnecessary energy expenditure [16,17].

The development of alternative strategies for reducing the negative effects of the harsh chemicals used in suppressing industrial microbial growth and biofilms formation is a necessity. One prophylactic approach is the pretreatment of surfaces with organic molecules that can later inhibit microbial adhesion [18,19]. Examples of low-toxicity biomolecules currently under investigation for their anti-biofilm activity include natural biosurfactants, such as surfactins and rhamnolipids [17,18,19]. The heterogeneous class of bioactive amines known as ‘alkaloids’ has, however, been largely overlooked in this context. A group of alkaloids derived from the venom of fire ants (Insecta: Hymenoptera: Formicidae: *Solenopsis*) trivially known as ‘solenopsins’ (reviewed in [20]) has been recently proposed as a potential candidate for biofilm inhibition [21] but remains mostly untested. In this context, the present report adds further information on the anti-biofilm effects from conditioning surfaces with solenopsin alkaloids obtained from solvent extraction of red imported fire ants.

## 2. Results

### 2.1. Solenopsins Extraction and Purification

The relative composition of solenopsin analogues obtained by hexane extraction of fire ants is given in Table 1 (and a chromatogram in Figure S1).

### 2.2. Antimicrobial Activity

**Figure 1:**
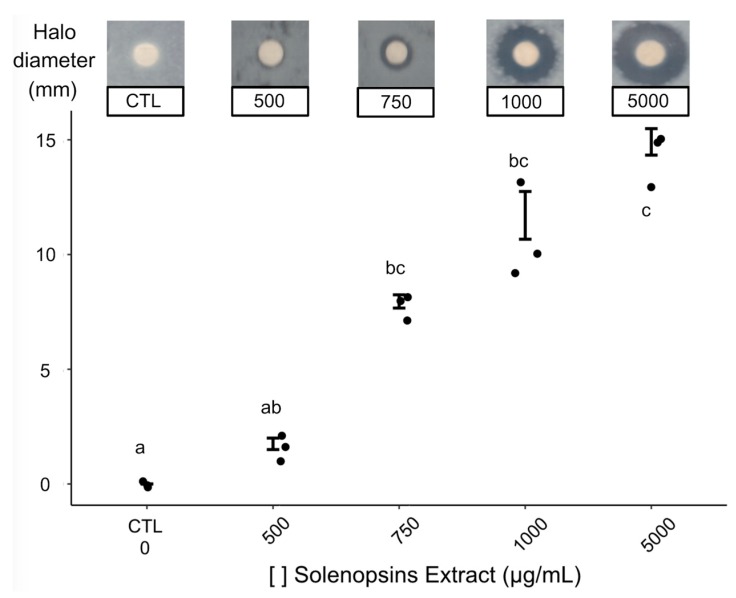
Diameters of inhibition zones resulting from the antimicrobial activity of solenopsins by the paper disk diffusion method. Points are raw data and vertical whiskers represent SD around the mean. Disks impregnated with different concentrations (μg/mL) of solenopsins were added to a confluent *Pseudomonas fluorescens* growth plate and incubated at 25 °C for 24 h. Treatments topped by same letters were statistically similar based on Dunn’s test at alpha = 0.05.

Obtained inhibition haloes against Petri dish-grown *P. fluorescens* were largest for the highest tested concentrations of solenopsins, providing evidence for antimicrobial activity (Figure 1). The solenopsin alkaloids presented antimicrobial activity at concentrations ranging from 750 to 5000 μg/mL, yielding mean diameters of inhibition halos between 8 and 14 mm (Figure 1). The minimum inhibitory concentration (MIC), estimated by log linear regression, was 370.4 μg/mL (Figure S2).

### 2.3. Effect of Preconditioning on Cell Adhesion and Biofilm Formation

**Figure 2:**
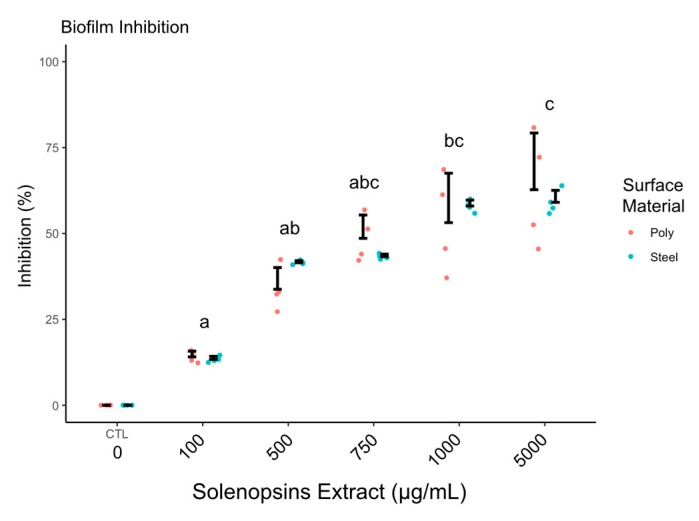
Inhibition of biofilm formation (as discounted % relative to control) by *Pseudomonas fluorescens* ATCC 13525 on surfaces of polystyrene and stainless steel 304 conditioned with solenopsin alkaloids at different concentrations. Points are raw data and vertical whiskers represent SD around the mean; ‘CTL’ stands for negative control and ‘poly’ for polystyrene. Treatments accompanied by same letters were statistically similar based on Dunn’s test at alpha = 0.05: no difference was observed between results with different surface materials.

Figure 2 illustrates anti-biofilm activity from polystyrene and stainless steel surfaces preconditioned with solenopsins. Conditioning yielded maximal inhibitions of cell adhesion of 80.7% on polystyrene and 63.9% on stainless steel under the concentration of 5000 μg/mL, although no significant difference is observed from 1000 μg/mL (*p*-values: 0.3318, 0.5000).

**Figure 3:**
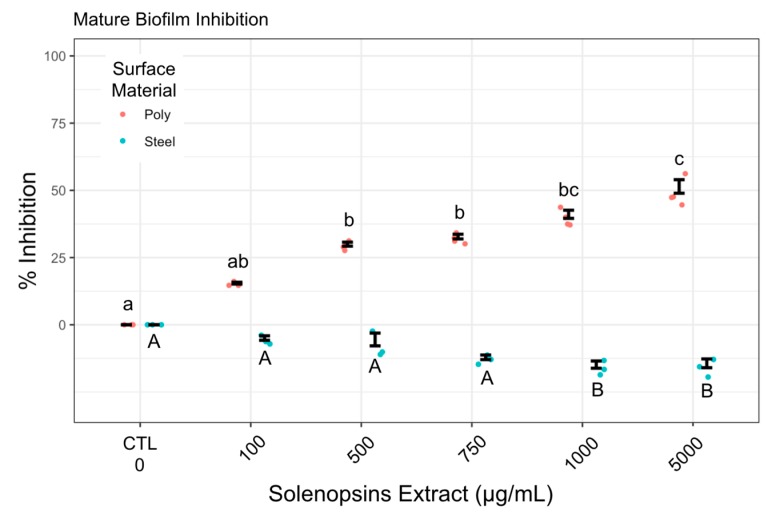
Reduction of mature biofilm (as % discounted of controls) by *Pseudomonas fluorescens* ATCC 13525 on non-conditioned surfaces of polystyrene (red) and stainless steel 304 (blue) by solenopsin alkaloids at different concentrations. Points are raw data and vertical whiskers represent SD around the mean; ‘CTL’ stands for negative control and ‘poly’ for polystyrene. Concentration groups within the same surface treatment accompanied by the same letter did not differ significantly by Dunn’s test at alpha = 0.05 (polystyrene: lowercase on top; stainless steel: uppercase below).

In addition to an antiadhesive effect, solenopsins had some capacity of reducing mature biofilms (i.e., preformed biofilm) growing on non-conditioned polystyrene (Figure 3, in red), though complete eradication was not observed. No effect was observed on mature biofilm grown on non-conditioned steel (Figure 3, in blue).

### 2.4. Quantification of Viable Cells

**Figure 4:**
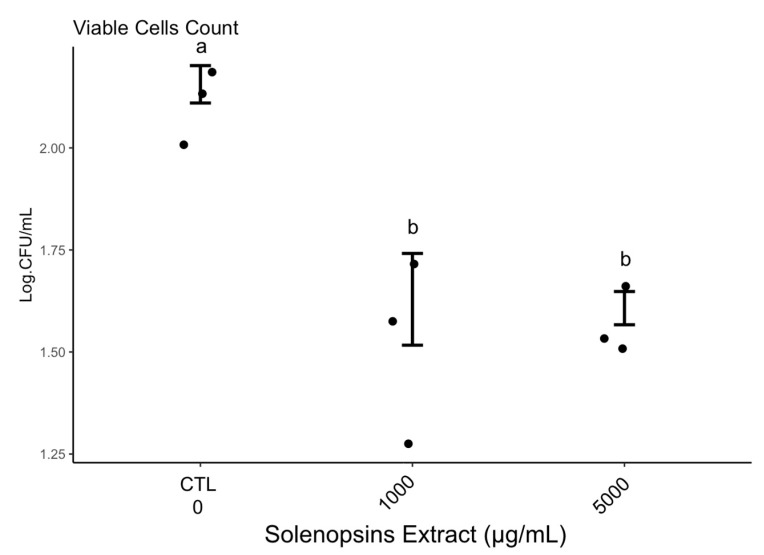
Viability of cells (in log/mL) recovered from biofilm of *Pseudomonas fluorescens* ATCC 13525 formed on surfaces of polystyrene conditioned with extracted solenopsins. No viable cells were recovered from a conditioned stainless steel coupon. Points are raw data and vertical whiskers represent SD around the mean; ‘CTL’ is negative control, and concentration groups topped by the same letter did not differ significantly by Dunn’s test at alpha = 0.05.

**Figure 5:**
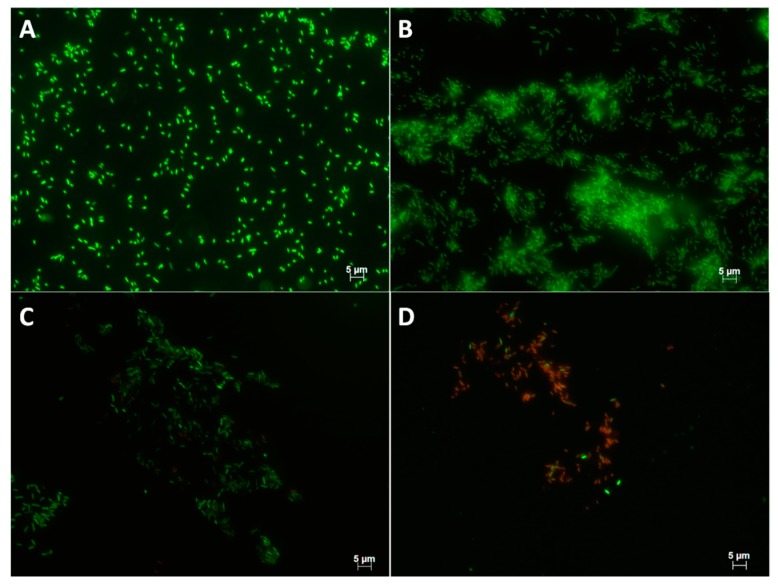
Surface adhesion by *Pseudomonas fluorescens* ATCC 13525 on polystyrene (left-hand panels) and stainless steel (right-hand panels) coupons after 24 h of incubation, as shown by epifluorescence of viable cells: Top panels (**A**,**B**) are negative non-conditioned controls; panels (**C**,**D**) are surfaces conditioned with 1000 µg/mL solenopsin alkaloids.

Colony-forming unit counts indicated a decrease of viable cells recovered from biofilm grown on surfaces conditioned with solenopsins (Supplementary Table; Kruskal–Wallis chi-squared = 12.3579, df = 4, *p*-value = 0.01) relative to non-conditioned controls (Figure 4). The number of viable cells recovered from biofilms grown on polystyrene conditioned with solenopsins was remarkably lower than in non-conditioned controls (Figure 4), and cells recovered from the conditioned steel surface were not viable (Table S1). The pattern observed was congruent with observations by epifluorescence microscopy (Figure 5). Controls had many layers of adhered cells (Figure 5A,B).

### 2.5. Physicochemical Properties: Surface Characteristics

Surface conditioning works by inhibiting microbial adhesion. This may be due to physicochemical surface alterations by amphiphilic (surface-active) compounds [19]. Therefore we tested the obtained solenopsins for a potential amphiphilic character, using the biosurfactant rhamnolipids as positive controls. Obtained contact angle measurements (Θ_w_) and energetic characteristics of polystyrene and stainless steel coupons conditioned with solenopsins and rhamnolipids are shown in Table S1. Non-conditioned polystyrene and stainless steel surfaces were shown as hydrophobic, and become more hydrophilic only from conditioning with rhamnolipids. Accordingly, as illustrated by results in Table S2, conditioning with solenopsins does not change the hydrophobic character of these surfaces.

## 3. Discussion

Surfaces made of polystyrene and stainless steel AISI 304 are widely employed in industrial and healthcare facilities, where they frequently come into contact with organic material. The formation of surface biofilms offers a permanent source of food and instrumental contamination. Currently, disinfectants and harsh chemicals are used in preventing industrial biofilms, with limited efficiency. For instance, the most commonly-employed sanitisers in Brazilian food facilities are industrial chemicals based on the following active principles: quaternary ammonium amines, active chlorine released from bleaches and NaDCC, organic peroxide from peracetic acid, inorganic peroxide from oxygenated water, and iodine solutions [22]. Milk processing lines worldwide employ a cleaning protocol called Cleaning-in-Place (CIP) that is based on a series of surface washing steps using water and disinfectants at high temperatures. However, a systematic assessment of CIP demonstrates it is inefficient in controlling biofilm formation (e.g., [23]) and will not completely remove attached bacteria [24].

New strategies to improve the efficiency of surface cleaning are continuously proposed. For example, biodegradable low-toxicity biosurfactant extracts from microbes have gathered considerable attention for their antimicrobial and anti-adhesive activities [17,18,19]. Also, a number of plant-derived alkaloids have been demonstrated to inhibit the formation of and disperse bacterial biofilms [25,26,27,28] that are often attributed to either intrinsic direct antimicrobial activity [29] or unknown effects [26,28]. Solenopsins are animal-derived alkaloids active against a number of microbes [20,30,31] that have been demonstrated to affect biofilm formation via a molecular disruption of quorum sensing in *Pseudomonas aeruginosa* (Schroeter 1872) [21].

No previous investigation on the potential of solenopsin alkaloids for surface conditioning has been published to date, although the possibility was raised by Fox [20]. Herein we demonstrate conditioning of polystyrene and stainless steel surfaces with solenopsins from *S. invicta* results in inhibited posterior microbial adhesion.

### 3.1. Compounds Production/Extraction

It should be noted that the venom alkaloids extraction method [32] resulted (Table 1) in considerable variation in the relative proportions of solenopsin B (aka ‘C13’) and solenopsins C (aka ‘C15’) in comparison to a typical venom profile of *S. invicta* workers (e.g., compare with patterns presented in [33]). Whether the obtained pattern was a natural alteration associated with uncontrolled variables such as environmental conditions and relative proportions of collected castes, or representative of a local cryptic species (e.g., as in [33,34]) is the subject of ongoing investigation.

The properties of natural extracts are defined by their relative chemical composition, which ultimately depends on extraction methods. This leaves a number of open questions such as: What are the effects of the isolated isomers and their relative contributions to the observed effects? Would different combinations (or relative proportions) of isomers differently affect biofilm formation? These questions are currently under investigation in parallel studies using synthetic solenopsin analogues.

There are a number of published methods for the extraction and purification of venom alkaloids [32,35,36] that enable obtaining gram-amounts of solenopsins in different degrees of purity. Minding that fire ants are a top-concern world invasive species [37], harvesting solenopsins from highly infested areas may be feasible for small-scale applications. Concerning artificial synthesis, despite several published methods, purchasing artificial solenopsins is restricted to few companies (e.g., WuXi AppTec of Shanghai, China) and can be prohibitively expensive for large-scale applications. Nonetheless, remaining obstacles for obtaining the compounds are likely circumventable by a sudden increase in market demand, particularly considering the number of other biotech applications proposed for solenopsin alkaloids (reviewed in [20]).

### 3.2. Antimicrobial Activity

Figure 1 clearly illustrates dose-dependent antimicrobial activity of solenopsins against biofilm-secreting *P. fluorescens.* Following a similar test, Jouvenaz et al. [31] found limited antimicrobial activity of synthetic solenopsins diluted 1:1000 against four out of 12 tested Gram-negative bacteria, namely *Shigella flexneri*, *Sh. boydii*, *Salmonella typhimurium,* and *Sa. paratyphi* (Table 1 of p. 292 in [31]). All the active solenopsin analogues tested by Jouvenaz et al. [31] exhibited inhibition haloes of 8 mm diameter or less, which are suggestive of a weaker effect than the observed in our study for the extract doses of 750 µg/mL and above (Figure 1). As discussed in the previous section, some augmented effect may have resulted from a natural combination of different solenopsin analogues, which awaits further evaluation using synthetic mixtures simulating natural venoms. Another study testing the antimicrobial activities of solenopsins extracted from *S. invicta* reported a growth inhibition halo of about 15 mm using 3800 μg/mL of solenopsins against the Gram-positive bacterium *Clavibacter michiganensis*; this was roughly the same inhibition halo diameter observed in our study against *P. fluorescens*, but with the higher concentration of 5000 μg/mL. It should be minded that Gram-positive bacteria such as *C. michiganensis* are reported to be more susceptible to the effects of solenopsins than Gram-negative bacteria like *P. fluorescens*, as observed by Sullivan et al. [38] and Jouvenaz et al. [31]. Further dedicated studies screening natural and synthetic solenopsins against a range of microorganisms are needed in order to elucidate their relative antibiotic potential and microbial resistance.

The mechanism of antimicrobial action of solenopsins has remained unclear, but according to Lind [39], they may induce a permeability change in the plasmatic membrane, promoting leakage of cellular components. The same authors have proposed an amphipathic nature of the molecules as the origin of this property. We have, however, found no evidence of chemical surface-activity for solenopsins based on pilot tests compared with detergent controls (Table S3).

Other alkaloids of plant origin are known to present a broad spectrum of activity against bacteria and fungi [2,40], including active inhibition of biofilm formation. For example, Robbers et al. [41] observed that benzophenantridine from the roots of the bloodroot *Sanguinaria canadensis* suppresses bacteria from causing dental plaques. Also, Pereira et al. [42] demonstrated that alkaloids from the pomegranate *Punica granatum* reduced the adhesion of five strains of *Streptococcus* to glass surfaces.

### 3.3. Biofilm Formation and Suppression

Microbial biofilms are secreted by surface-adhered microorganisms. Therefore, inhibition of biofilm formation can be achieved by preventing microbial adhesion. A long-term strategy for protecting exposed surfaces against microbial adhesion is known as surface conditioning, which is based on the adsorption of antimicrobial or anti-adhesive molecules [19]. Preconditioning with solenopsin alkaloids inhibited the formation of biofilm regardless of the treated surfaces material (Figure 2). Lind et al. [39] suggested that solenopsins would have an amphipathic character, which might account for a change in surface physicochemical characteristics resulting in biofilm growth inhibition, as seen with natural detergents such as rhamnolipids and surfactins [17,18,19]. However, as discussed further in Section 3.5, the surface conditioning effects are not related to some detergent-like amphipathic chemical character of solenopsins, contrary to the proposed by Lind [39].

The effects of solenopsins on biofilms preformed on non-conditioned exposed surfaces (i.e., ‘mature’ biofilms) were also evaluated. Exposure of mature biofilms to solenopsins somehow resulted in the elimination of up to 56% of the biofilm from a polystyrene surface in a dose-dependent manner (Figure 3). However, on stainless steel surfaces, exposure to solenopsins yielded no reduction, and may have been accompanied by a small increase in secreted biofilm, which could be indicative of a counter-synergistic effect. This possibility deserves deeper investigation. A previous study by Park et al. [21] demonstrated that exposure to synthetic solenopsin A (aka ‘C11’, Table 1) dissolved in growth medium will inhibit biofilm formation by *P. aeruginosa* on non-conditioned surfaces via quorum sensing signaling interference.

### 3.4. Quantification of Viable Cells

Cell counts demonstrate a sharp reduction in the density of viable cells on polystyrene surfaces conditioned with solenopsins (Figure 4, Table S1). The same general pattern is confirmed by epifluorescence microscopy (Figure 5). No viable or cultivable cells were recovered from the matrix grown on conditioned steel (Table S1), which is suggestive of a stronger activity of the compounds when applied to the metal. This phenomenon warrants deeper investigation.

### 3.5. Physico-Chemical Tests for Surfactant Chemistry

The solenopsin alkaloids were tested to investigate their potential chemical characteristics as surfactants (surface-active compounds), since detergent-like (amphipathic properties) were suggested by some authors (e.g., Lind [39], pers. comm. of Dhammika Nanayakkara) as the likely mode of action for the antimicrobial activity of solenopsins. Amphipathic compounds physically interact by decreasing interfacial tension, thus leading to the formation of micelles, microemulsions, and adsorption to available surfaces. In theory, this chain of physicochemical processes could interfere with the microbial adhesion and biofilm formation [43,44].

According to Vogler [42], the degree of hydrophobicity is measured by the contact angle to water, where contact angles below 65° are indicative of hydrophilic surfaces. A surface is considered hydrophilic when the value of ΔGiwi (total free energy) is positive and Θ_w_ is less than 65°, and hydrophobic when the value of ΔGiwi is negative and Θ_w_ is greater than 65° [45,46,47]).

Conditioning with rhamnolipids has been described as affecting surface hydrophobicity [48,49] and the formation of bacterial biofilms [17]. Accordingly, the obtained rhamnolipids extract displayed remarkable tensioactive effects, illustrated by observed reduction in water surface tension. On the other hand, the solenopsins extract did not display any effect on water surface tension, possibly because of their apolar character, as these alkaloids are described to be largely insoluble in water [20]. Therefore, the mechanisms of biofilm inhibition by these alkaloids must be markedly different from that of biosurfactants, and not related to amphipathic chemistry.

## 4. Conclusions

The solenopsins extract exhibited potential application as a surface conditioning agent while acting as an antibiotic against biofilm formation. This class of compounds provides novel tools for the development of sustainable strategies to prevent or reduce biofilms on surfaces with potential application in different structural systems. Ongoing and future tests will provide further information into their modes of action, particularly for the solenopsin alkaloids of which so little is known.

## 5. Materials and Methods

### 5.1. Solenopsins Extraction and Purification

Nests of the fire ant *S. invicta* were collected from the university campus of the Federal University of Rio de Janeiro, Brazil, from which ants and their venom alkaloids were sequentially extracted following procedures detailed in [29]. Species identification was confirmed based on the presence of a clear frontal streak and a well-developed medial clypeal tooth [50]; voucher specimens were deposited at Museu Nacional do Rio de Janeiro (MNRJ). For venom purification, the obtained ants were immersed in a biphasic hexane/water (1:5) mixture, from which the organic phase was collected and passed through a glass column containing ca. 10 g of silica MESH 200–400 (Sigma, Mendota Heights, MN, USA). The column was washed three times with hexane (Merck, Kenilworth, NJ, USA, purity 98%), and finally the alkaloids were eluted with pure acetone (Merck, Kenilworth, NJ, USA, purity 99.8%). Obtained alkaloids were concentrated under a N_2_ flux, and weighed with analytical scales.

The isomeric proportions of solenopsins were determined by a Gas Chromatography system coupled with Mass Spectrometry (GCMS, QP2010A Shimadzu, Rio de Janeiro, Brazil) according to procedures detailed in Fox et al. [51] (also see Supplementary Files). Obtained chromatogram peaks were identified as also described in [51] and tentatively quantified by relative area to an external standard (nicotine from Sigma-Aldrich, Mendota Heights, MN, USA) at 0.5 µg/µL using OpenChrom v. 1.1.0 (University of Hamburg, Hamburg, Germany).

### 5.2. Microbial Tests

Antimicrobial tests were performed with *Pseudomonas fluorescens,* which is a safer alternative to testing with the more ubiquitous pathogen *Pseudomonas aeruginosa* [6]. Stocked *P. fluorescens* cultures were inoculated in Petri dishes containing nutrient agar and incubated at 25 °C for 24 h. After this period, standard bacterial suspensions were adjusted to a concentration of 10^9^ colony forming units (CFU)/mL, according to [18].

#### 5.2.1. Antimicrobial Activity

Antimicrobial activity was assessed by the method of disc diffusion as described in [52], and the minimum inhibitory concentration (MIC) was estimated by logarithm linear regression [53]. Suspensions of 10^6^ CFU/mL of *P. fluorescens* were obtained by serial dilutions, estimated by a spectrophotometer at 600 nm wavelength to a final volume of 100 μL, and spread on a Petri dish containing nutrient agar. Sterile filter paper discs (Whatman 3) of 6 mm diameter were immersed in the different concentrations of solenopsins (500, 750, 1000, and 5000 μg/mL) in ethanol. Negative controls had pure ethanol. Following solvent evaporation, paper discs were placed at the centre of the inoculated Petri dishes, which were incubated at 25 °C for 24 h. The diameter of the inhibition halo was measured from the rim of the paper disc using a caliper. Four independent replications were carried out.

#### 5.2.2. Quantification of Biofilm Formation

Biofilm formation was induced by inoculating 20 µL aliquots of standardised suspensions from Section 5.2.1. into 96-well microplates made of either polystyrene (OLen from Kasvi, São José do Pinhais, Brazil) or stainless steel (AISI 304) containing a nutrient broth. These microplates were kept at 25 °C for different time periods to perform biofilm formation kinetics. At the end of each time period, the growth medium was removed, wells were carefully washed with distilled water, fixed for 15 min with methanol (Merck, São Paulo, Brazil; 99.9% purity), and stained for 20 min with 1% (*w/v*) crystal violet after [54]. Finally, the optical density at 570 nm of the stained solution was measured as an estimation for overall cell adhesion following Stepanovic et al. [55]. Mean values are presented from four independent experiments.

#### 5.2.3. Effect of Surface Conditioning on Cell Adhesion

Microplates, as described in Section 5.2.2, were surface-conditioned according to Nitschke et al. [19]. Surface conditioning solutions of solenopsins were prepared in ethanol (Merck, São Paulo, Brazil; 99.5% purity) at the concentrations of 0, 100, 500, 750, 1000, or 5000 µg/mL. After 24 h of conditioning by immersion, the plate surfaces were washed with sterile distilled water and left to dry at room temperature. Cell adhesion was estimated with crystal violet, as described in Section 5.2.2.

#### 5.2.4. Effect of Surface Conditioning on Cell Viability

Polystyrene and stainless steel surfaces were conditioned as described in Section 5.2.3 and inoculated with a suspension of 10^9^ CFU/mL of *P. fluorescens*. Following biofilm formation, surfaces were washed to remove any non-adhered cells, and biofilms were scraped off for analysis. The proportion of viable cells recovered from biofilms growing on conditioned surfaces were estimated by the plate spreading technique. A 100-μL aliquot per sample was spread using a sterile Drigalski loop onto a Petri dish containing nutrient agar. Dishes were incubated at 25 °C for 24 h, and the resulting CFU were visually counted.

#### 5.2.5. Epifluorescence Microscopy Observations

Plate coupons (2 cm^2^) of polystyrene and stainless steel were conditioned as described in Section 5.2.3 with 1 mg/mL solenopsins for 24 h at 25 °C, and immersed in a nutrient broth containing 10^9^ CFU/mL *P. fluorescens.* These surfaces were incubated at 25 °C for 20 h and 16 h, respectively, washed with distilled water to remove non-adhered cells, and stained with the bacterial cell viability fluorescent marker L7012 LIVE/DEAD^®^ Baclight^TM^ (Molecular Probes Inc., Eugene, OR, USA) according to the manufacturer’s manual. Finally, the treated surfaces were observed under a Zeiss Axioplan 2 microscope (Oberkochen, Germany) equipped with an epifluorescence system under excitation/emission wavelengths of 480/500 nm for SYTO 9 and 490/635 nm for propidium iodite. Pictures were taken with a digital camera Color View XS (AnalySIS GmBH, Karlsruhe, Germany).

### 5.3. Physico-Chemical Tests for Surfactant Chemistry

The obtained solenopsins extract was subjected to physicochemical assays to test for a potential amphiphilic character. The natural biosurfactants known as rhamnolipids—well-known amphiphilic compounds with anti-biofilm activity [17,49,56]—were used as positive controls.

#### 5.3.1. Extraction of Biosurfactants (Positive Controls)

Rhamnolipid production from *P. aeruginosa* was induced by procedures described elsewhere [57]. Cells were removed by centrifugation, and the supernatant containing the compounds was sterilised, filtered (pore size 0.45 µm), and stored at 4 °C until use. The supernatant was acidified with 1.0 N HCl to pH 3.5 and directly extracted with ethyl acetate 1:3, from which the organic phase was recovered, and incubated with anhydrous sodium sulfate to remove water residues. The extract was recovered from the solvent using a rotary evaporator, redissolved in methanol, and finally lyophilised. The obtained extract was partitioned with methanol: chloroform: 2-propanol in the ratio of 1:2:4 with 7.5 mM ammonia acetate, and finally centrifuged at 12,000× *g* for 5 min to remove impurities.

#### 5.3.2. Physicochemical Properties

The obtained crude extracts of solenopsins and rhamnolipids were subjected to analyses of surface tension (ST) and critical micellar concentration (CMC). These physicochemical properties were estimated based on the pendant drop technique on a drop shape analyser (DSA 100S Model OF3210) following [58,59]. Measurements for ST and CMC were taken from n = 10 drops at 23 °C and 55% relative humidity.

#### 5.3.3. Surface Characteristics

Small coupons (2 cm^2^) of either polystyrene or stainless steel grade 304 were cleaned as in [19], and conditioned with either rhamnolipids or solenopsins. The conditioned surfaces were washed with distilled water and left to dry at room temperature. Finally, the sessile drop method described in Section 5.3.2. was used to measure the contact angle between the surface and 7 µL droplets of distilled water, formamide, and ethylene glycol using a goniometer. The angles were measured as described in [46] at 23 °C and 55% relative humidity.

The surface free energy obtained from the contact angle was calculated from the surface tension components of each tested liquid according to the equation below (as in Van Oss et al. [60]):

$$\left(1+cos\theta \right)\gamma_{i}^{TOT}=2\left[\left(\sqrt{\gamma_{s}^{LW}\gamma_{i}^{LW}}\right)+\left(\sqrt{\gamma_{s}^{+}\gamma_{i}^{-}}\right)+\left(\sqrt{\gamma_{s}^{-}\gamma_{i}^{+}}\right)\right]$$

where *θ* is the contact angle between the liquid and the surface, *γ^TOT^* is the total surface free energy, *γ^LW^* is the Lifshitz-van der Waals component, *γ^AB^* is the Lewis acid-base property, and *γ^+^* e *γ*^−^ are the electron acceptor and donor components, respectively; *γ^TOT^ = γ^LW^ + γ^AB^* and *γ^AB^* = $2\sqrt{\gamma^{+}\gamma^{-}}$.

Surface hydrophobicity was determined by contact angle measurements using the approach of Van Oss et al. [60] and Van Oss [46]. The results were calculated according to the equation below:

$$\Delta Giwi=\;-2\left(\sqrt{\gamma_{l}^{LW}-\gamma_{w}^{LW}}\right)\;-4\left(\sqrt{\gamma_{l}^{+}\gamma_{w}^{-}}+\sqrt{\gamma_{w}^{-}\gamma_{l}^{+}}-\sqrt{\gamma_{l}^{+}\gamma_{l}^{-}}-\sqrt{\gamma_{w}^{+}\gamma_{w}^{-}}\right)$$

#### 5.3.4. Hydrophilic-Lipophilic Balance (HLB)

The HLB value indicates whether a surfactant will promote water-in-oil or oil-in-water emulsions. According to Griffin [61], the HLB can be calculated as:

HLB = 20 × (MWHP/MWSA)

where MWHP is the molecular weight of the hydrophilic part and MWSA is the molecular weight of the whole surface-active agent, giving a result on a scale of 0 to 20. An HLB value of 0 corresponds to a completely lipophilic/hydrophobic molecule, and a value of 20 corresponds to a completely hydrophilic/lipophobic molecule. The HLB value can be used to predict the surfactant properties of a molecule (see Table S2).

### 5.4. Statistics

All analyses and plots were generated with R v. 3.0.0 using ‘ggplot2’ and packages ‘ddply’ and ‘reshape2.’ Raw data, as well as plotting and analytical scripts, are provided as Supplementary Materials. Results were analysed non-parametrically using Wilcoxon–Mann–Whitney (2-factorial analyses) or Kruskal–Wallis (3-factorial analyses) tests. Equivalent results are obtained by parametric counterparts (not shown).

## Figures and Tables

**Table 1:** Composition of solenopsins from a whole-nest extraction of the fire ant *Solenopsis invicta.*

Compound Name	Short Trivial Name	Chemical Formula	Diagnostic Ions-*m/z*	RT (Initial)	Relative Abundance (Area %)
*cis*-2-Me-6-Tridecyl-Piperidine	C13	C_19_H_39_N	280 (M^+^), 266, 98	19.942	2.594
*trans*-2-Me-6-Tridecenyl-Piperidine	C13:1	C_19_H_38_N	279 (M^+^), 264, 180, 124, 111, 98	20.167	76.528
*trans*-2-Me-6-Tridecyl-Piperidine	C13	C_19_H_39_N	280 (M^+^), 266, 98	20.375	6.349
*cis*-2-Me-6-Pentadecyl-Piperidine	C15	C_21_H_43_N	309 (M^+^), 308, 294, 98	21.550	0.394
*trans*-2-Me-6-Pentadecenyl-Piperidine	C15:1	C_21_H_42_N	307 (M^+^), 292, 228, 154, 124, 111, 98	21.833	11.554
*trans*-2-Me-6-Pentadecyl-Piperidine	C15	C_21_H_43_N	309 (M^+^), 308, 294, 98	22.025	2.580

## Supplementary Materials

The following are available online at https://www.mdpi.com/2072-6651/11/7/420/s1, Figure S1: Representative chromatogram of venom alkaloids extracted with hexane, Figure S2: Linear regression from mean obtained inhibition halos from disk-diffusion using different concentrations (μg/mL) of solenopsins added to a confluent Pseudomonas fluorescens growth plate, incubated at 25 °C for 24 h, Table S1: Viability of cells recovered from 1 mL of biofilm of *Pseudomonas fluorescens* formed on surfaces of polystyrene or stainless steel conditioned with venom solenopsins extracted from the fire ant *Solenopsis invicta*, Table S2: Values of surfactants and their respective applications (Adapted from [54]), Table S3: Physicochemical properties of surfaces conditioned with rhamnolipids extracted from *Pseudomonas aeruginosa* and venom solenopsins extracted from red imported fire ants *Solenopsis invicta*; R scripts used for statistical analyses and generating figures, including raw data; Raw chromatogram GM-MS data file of obtained solenopsins, Raw GC-MS method settings file.
