# Supplementary material for: Fire Ant Venom Alkaloids Inhibit Biofilm Formation
Source: Toxins (Basel). 2019 Jul 18;11(7):420. doi: 10.3390/toxins11070420 (PMC6669452; doi:10.3390/toxins11070420)
Supplement: Supplementary file 1 [file toxins-11-00420-s001.zip › toxins-518897 final supple/toxins-518897 proof supplementary.docx]

Supplementary Materials: Fire Ant Venom Alkaloids Inhibit Biofilm Formation

Danielle Bruno de Carvalho, Eduardo Gonçalves Paterson Fox, Diogo Gama dos Santos,
Joab Sampaio de Sousa, Denise Maria Guimarães Freire, Fabio C. S. Nogueira,
Gilberto B. Domont, Livia Vieira Araujo de Castilho and Ednildo de Alcântara Machado


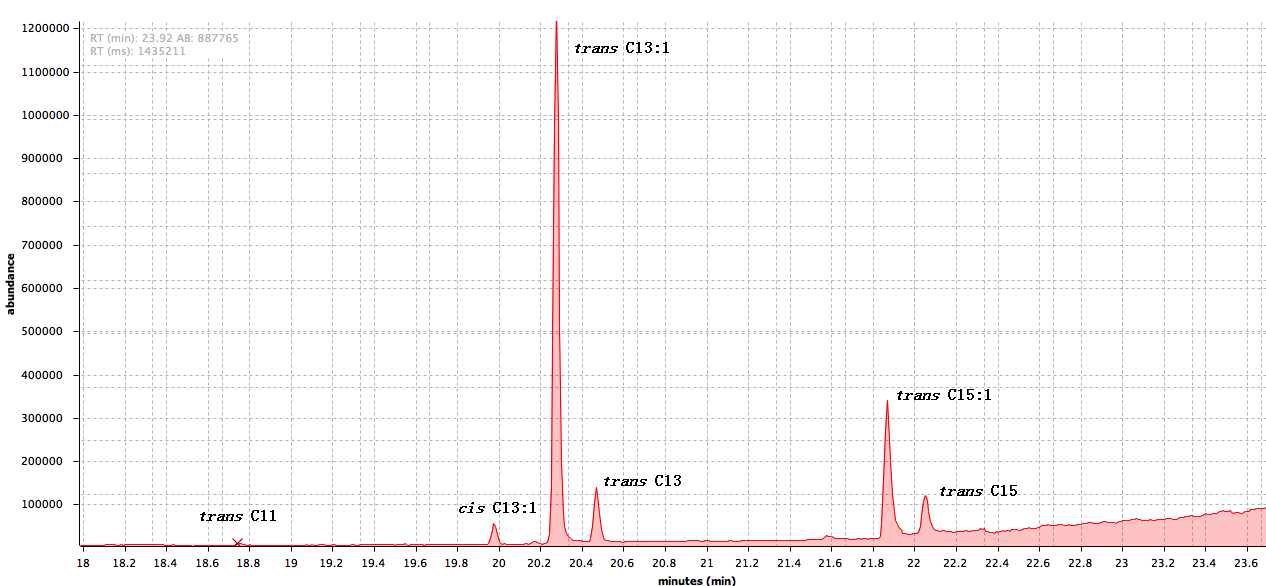


**Figure S1.** Representative chromatogram of venom alkaloids extracted with hexane: acetone elution as described in [32], with peaks identified as described in [34], from red imported fire ants *Solenopsis invicta* collected in Rio de Janeiro, Brazil.


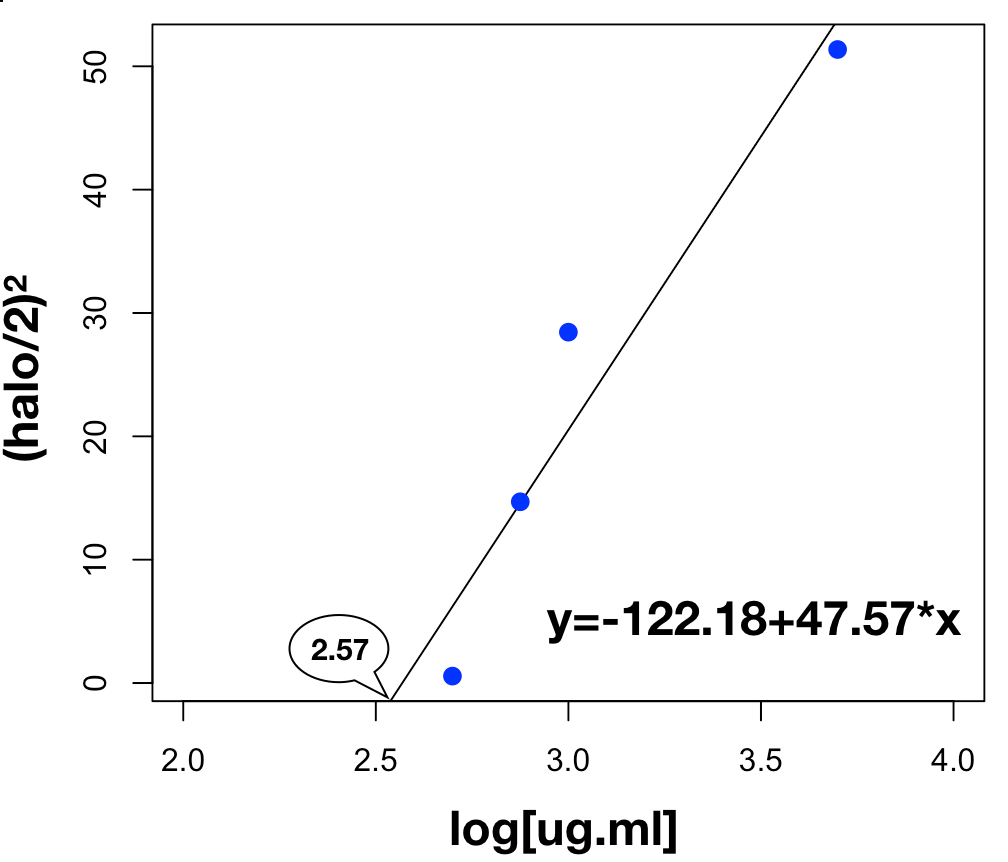


**Figure S2.** Linear regression from mean obtained inhibition halos from disk-diffusion using different concentrations (μg/mL) of solenopsins added to a confluent *Pseudomonas fluorescens* growth plate, incubated at 25 °C for 24 h. Same results as presented in Figure 1, herein transformed for MIC estimation (at x = 0, where indicated) according with [53].

**Table 1.** Viability of cells recovered from 1 mL of biofilm of *Pseudomonas fluorescens* formed on surfaces of polystyrene or stainless steel conditioned with venom solenopsins extracted from the red imported fire ant *Solenopsis invicta*.

| **Treatments** | **Polystyrene** | **Stainless Steel** |
| --- | --- | --- |
|  | **Viable Cells** | **Viable Cells** |
| Control  0 µg/mL | 130.00 ± 26.53 *a* | 155.00 ± 35.16 |
| 1000 µg/mL | 36.00 ± 16.38 *b* | Not detected |
| 5,000 µg /mL | 37.00 ± 7.31 *b* | Not detected |

Means followed by SD (*N* = 3); different letters within the same column indicate statistically different values by Kruskal-Wallis followed by Dunn’s test at alpha = 0.05; values between rows (materials) did not differ by Wilcoxon’s rank test at alpha = 0.05 (W = 105, p-value = 0.7714).

**Table S2.** Values of surfactants and their respective applications (Adapted from [54]).

| **HLB scale** | **Use** |
| --- | --- |
| 4-6 | W/O emulsifiers |
| 7-9 | Wetting agents |
| 8-18 | O/W emulsifiers |
| 13-15 | Detergents |
| 15-18 | Solubilizers |

Notes: HLB - Hydrophilic-lipophilic balance; W - water; O – oil.

**Table S3.** Physicochemical properties of surfaces conditioned with rhamnolipids extracted from *Pseudomonas* *aeruginosa* and venom solenopsins extracted from red imported fire ants *Solenopsis invicta.*

| **Surface** | **Treatment** | **Ɵ_W_(degrees)** | **ΔGiwi (mJ/m^2^)** | **Ɣ ^LW^ (mJ/m^2^)** | **Ɣ ^AB^ (mJ/m^2^)** | **Ɣ ^+^ (mJ/m^2^)** | **Ɣ ^−^ (mJ/m^2^)** |
| --- | --- | --- | --- | --- | --- | --- | --- |
| Polystyrene | Control (H_2_O) | 66.7 ± 0.3 | −51.0 ± 1.7 | 46.9 ± 1.7 | 6.9 ± 0.5 | 3.5 ± 0.3 | 3.5 ± 0.3 |
|  | RL | 10.7 ± 1.2 | −30.0 ± 0.5 | 79.2 ± 0.2 | 18.4 ± 0.1 | 9.2 ± 0.1 | 9.2 ± 0.1 |
|  | Control (EtOH) | 64.4 ± 0.9 | −57.4 ± 0.8 | 50.1 ± 0.7 | 5.5 ± 0.0 | 2.7 ± 0.0 | 2.7 ± 0.0 |
|  | Sol | 63.3 ± 1.4 | −24.8 ± 0.7 | 51.6 ± 0.6 | 21.1 ± 0.6 | 10.6 ± 0.3 | 10.6 ± 0.3 |
| Stainless Steel 304 | Control (H_2_O) | 74.3 ± 0.8 | −76.0 ± 5.1 | 58.0 ± 1.6 | 2.3 ± 0.4 | 1.1 ± 0.1 | 1.1 ± 0.2 |
|  | RL | 7.9 ± 0.6 | −32.5 ± 1.3 | 80.7 ± 0.6 | 18.2 ± 0.2 | 9.1 ± 0.1 | 9.1 ± 0.1 |
|  | Control (EtOH) | 60.5 ± 0.3 | −58.0 ± 0.5 | 51.5 ± 0.4 | 5.2 ± 0.1 | 2.6 ± 0.1 | 2.6 ± 0.1 |
|  | Sol | 62.7 ± 1.7 | −58.4 ± 0.3 | 59.8 ± 0.2 | 5.6 ± 0.7 | 2.8 ± 0.3 | 2.8 ± 0.3 |

Means followed by SD (*N* = 4); Water contact angle - Ɵ_W;_ Surface hydrophobicity - ΔG^LW^_;_ Lifshitz-van der Waals component - Ɣ^LW^_;_ Lewis acid-basic properties - Ɣ^AB^_;_ electron donor component - Ɣ^-^ and electron acceptor component – Ɣ^+^; RL - Rhamnolipids; Sol –Solenopsin alkaloids.
